# Effect of previous utilization and out-of-pocket expenditure on subsequent utilization of a state led public-private partnership scheme “*Chiranjeevi Yojana*” to promote facility births in Gujarat, India

**DOI:** 10.1186/s12913-017-2256-6

**Published:** 2017-04-25

**Authors:** Sandul Yasobant, Hemant Deepak Shewade, Kranti Suresh Vora, Kristi Sidney Annerstedt, Petros Isaakidis, Nishith B. Dholakia, Dileep V. Mavalankar

**Affiliations:** 1 Indian Institute of Public Health Gandhinagar (IIPHG), Gandhinagar, India; 2 International Union Against Tuberculosis and Lung Disease (The Union), South-East Asia Office, New Delhi, India; 3 Karolinska Institutet, Stockholm, Sweden; 4 Médecins Sans Frontières (MSF)/Doctors Without Borders, Mumbai, India; 5 Department of Health & Family Welfare, Government of Gujarat, Gandhinagar, India

**Keywords:** Chiranjeevi Yojana (CY), Out of pocket expenditure (OOPE), Utilization, Facility birth, Gujarat, India

## Abstract

**Background:**

In Gujarat, India, a state led public private partnership scheme to promote facility birth named *Chiranjeevi Yojana (CY)* was implemented in 2005. Institutional birth is provided free of cost at accredited private health facilities to women from socially disadvantaged groups (eligible women). CY has contributed in increasing facility birth and providing substantially subsidized (but not totally free) birth care; however, the retention of mothers in this scheme in subsequent child birth is unknown. Therefore, we conducted a study aimed to determine the effect of previous utilization of the scheme and previous out of pocket expenditure on subsequent child birth among multiparous eligible women in Gujarat.

**Methods:**

This was a retrospective cohort study of multiparous eligible women (after excluding abortions and births at public facility). A structured questionnaire was administered by trained research assistant to those with recent delivery between Jan and Jul 2013. Outcome of interest was CY utilization in subsequent child birth (Jan–Jul 2013). Explanatory variables included socio-demographic characteristics (including category of eligibility), pregnancy related characteristics in previous child birth, before Jan 2013, (including CY utilization, out of pocket expenditure) and type of child birth in subsequent birth. A poisson regression model was used to assess the association of factors with CY utilization in subsequent child birth.

**Results:**

Of 997 multiparous eligible women, 289 (29%) utilized and 708 (71%) did not utilize CY in their previous child birth. Of those who utilized CY (*n* = 289), 182 (63%) subsequently utilized CY and 33 (11%) gave birth at home; whereas those who did not utilize CY (*n* = 708) had four times higher risk (40% vs. 11%) of subsequent child birth at home. In multivariable models, previous utilization of the scheme was significantly associated with subsequent utilization (adjusted Relative Risk (aRR): 2.7; 95% CI: 2.2–3.3), however previous out of pocket expenditure was not found to be associated with retention in the CY scheme.

**Conclusion:**

Women with previous CY utilization were largely retained; therefore, steps to increase uptake of CY are expected to increase retention of mothers within CY in their subsequent child birth. To understand the reasons for subsequent child birth at home despite previous CY utilization and previous zero/minimal out of pocket expenditure, future research in the form of systematic qualitative enquiry is recommended.

## Background

Of the total health expenditure in India, out-of-pocket payments contribute 51%, the Government contributes 33% and minor proportion is paid by third party such as insurance companies [1]. High out-of-pocket expenditure (OOPE) makes health services, including care for childbirth, inaccessible for the under privileged population especially the poor [2, 3]. Research has shown that women’s uptake of maternal health care services remains strongly associated with wealth, and high financial costs are considered a major barrier in maternal health care utilization [4–7].

Against this background, Governments and donors are exploring ways to reduce cost barriers for pregnant women and many demand-side financing schemes, designed to stimulate demand for maternal health care, have been implemented globally. One such scheme in India to promote institutional deliveries at public health facilities is “*Janani Suraksha Yojana (JSY or safe motherhood program)*”implemented nationwide. It is a conditional cash transfer scheme where the mother is paid a fixed amount after giving birth in a public health facility [6, 8]. However after a decade of the implementation of the scheme, OOPE remains high for child birth, which is associated delivery cost such as transportation, food, baby expenses [9, 10]. Instead, delivery services at public health facilities were often perceived as inferior to services offered at private facilities, and an increase in income was often accompanied by a preference for private health care [11].

To promote institutional birth, the Government of Gujarat implemented a state led public-private-partnership scheme called “*Chiranjeevi Yojana* (CY)” in the year 2005. “*Chiranjeevi Yojana”* in vernacular language means a scheme to live long. The scheme provides institutional birth free of cost in accredited private health facilities to women from socially disadvantaged groups [women living in households with income below the poverty line (BPL) and scheduled tribes (ST)] [12].

To date there are several studies on various aspects of CY including its utilization [13–19]. In our previous study on demand side utilization of CY, we found that more than half of the eligible beneficiaries still delivered either at home or at non-accredited private facilities [19]. Though CY beneficiaries received substantially subsidized delivery care compared to women who delivered in non-accredited private facilities, the services were still not completely free as was envisaged by the scheme [13, 19]. Incurring OOPE may be one of the important factors for subsequent utilization [13, 19].

Retention of mothers in this scheme in subsequent child birth is unknown. It would be pertinent for the state to know how many continue with subsequent CY utilization after previous CY utilization and the factors associated with it. Therefore, the present study aimed to determine the effect of previous utilization and previous OOPE on subsequent utilization among eligible CY beneficiaries in Gujarat, India. Specific objectives were to determine the i) number (proportion) with previous CY utilization stratified by subsequent CY utilization; ii) median OOPE in previous child birth stratified by subsequent CY utilization; iii) factors associated (including previous utilization and OOPE) with subsequent CY utilization.

## Methods

### Study design

This retrospective cohort study was part of a large scale community-based Maternal Health India (MATIND) project aimed to study the CY scheme in the state of Gujarat [20]. A subset of data belonging to multiparous eligible beneficiaries was included.

### Study setting

#### General setting

Gujarat state (population 60.4 million) is comprised of 33 districts, the average population of a district is two million. Districts are further divided into 10–20 blocks (sub-districts) of approximately 100,000 to 200,000 people. These districts have varying human development indices and different population compositions [21, 22]. The population is divided into socio-economic sub groups. Government of India uses the terms ‘Schedule Tribe (ST)’ to denote these traditionally marginalized populations. In addition the term ‘below poverty line (BPL)’ is also used to denote economically disadvantaged families. This documentation is used to avail special benefits under various programs of positive favorable action, CY scheme being one of them.

#### CY scheme

The government pays Indian Rupee ₹4000 per child birth (approximately USD$ 67) directly to the provider, irrespective of its type i.e. normal or operative (assisted or Caesarian section). Eligible beneficiaries have to visit the accredited CY facility within district with eligibility documentation to utilize the CY services free of cost. As of now, about 1 million births have been covered under CY [12, 15, 18].

### Study site

**Fig. 1:**
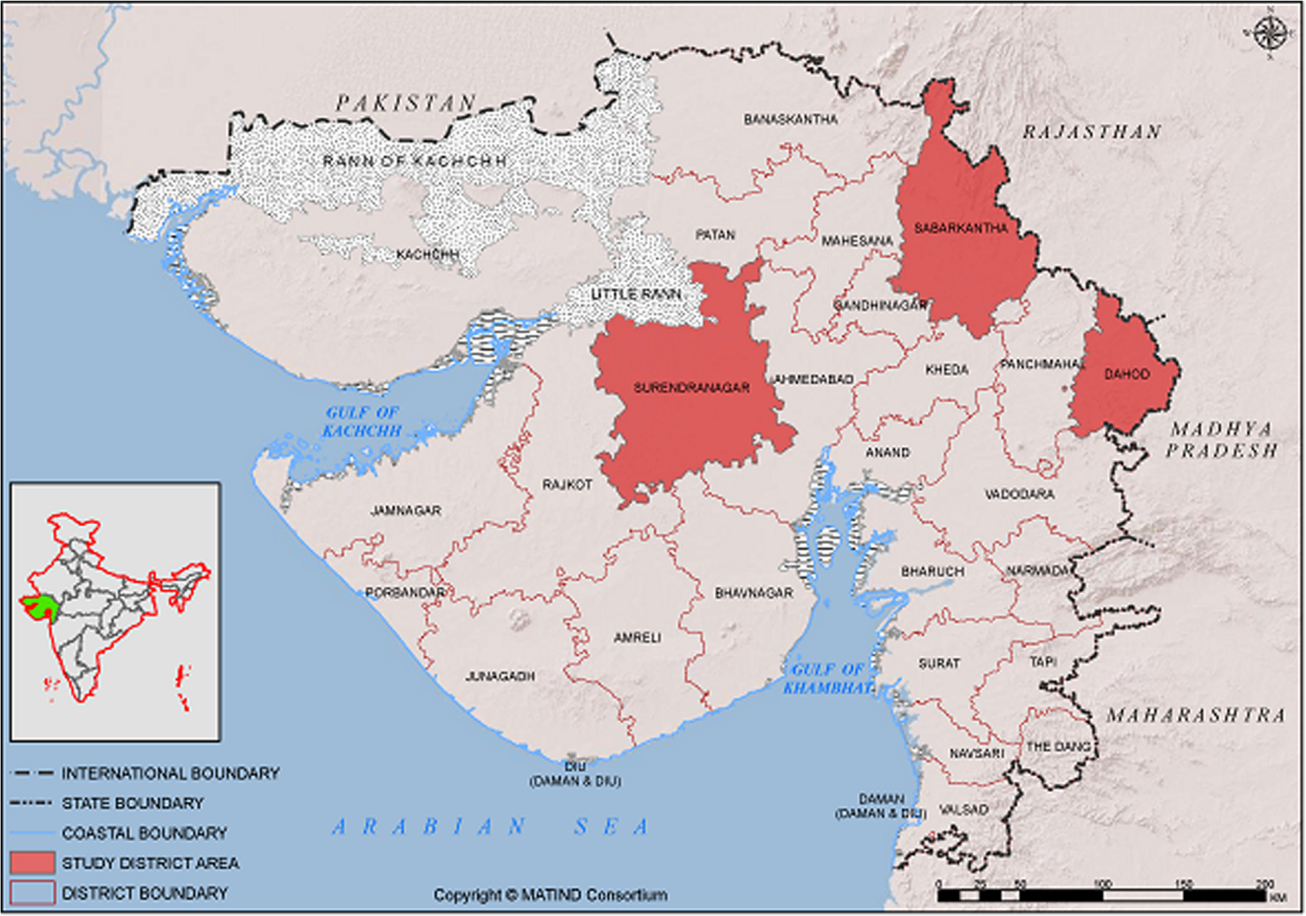
Map* of Gujarat (India) indicating the study districts (Dahod, Sabarkantha, Surendranagar). (*This map is a product of MATIND consortium and MATIND owns it’s copyright. Authors have written consent from MATIND consortium to use and adapt this map)

Community-based MATIND survey was conducted in three heterogeneous districts from the western, central and eastern belts of the Gujarat state (Sabarkantha, Surendranagar and Dahod) [23] (Fig. 1). The detail indicators of these sampled districts are shown in Appendix 1.

### Study population and sampling

The study population included eligible women in the study districts who delivered between July 2013 and November 2014. As CY providers were not evenly distributed geographically throughout the district, blocks in each district were selected to represent areas where there were no, low (one or two providers) or high number (more than two per block) of CY providers. In brief, in each of the 3 districts, 3–5 blocks were selected purposively. A list of all the villages in these blocks was compiled using the criteria: village population more than 1,000 and less than 2,500, greater than 40% BPL population and scattered all over the block. From the list, 142 villages were selected randomly to cover approximately 300,000 populations. The sampling methodology of MATIND survey has been described elsewhere [23].

**Fig. 2:**
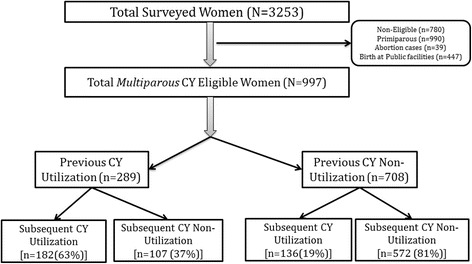
Chiranjeevi Yojana (CY)* utilization among eligible women who delivered between Jan-Jul 2013 in three districts of Gujarat, India (N = 997).(*CY, a state led public private partnership scheme to promote institutional birth among socially disadvantaged group)

The study population included multiparous eligible beneficiaries in the study districts that were under the community-based MATIND survey [20, 23] after excluding abortions and births in public health facility. Of 3253 women surveyed in MATIND, 2473 were eligible beneficiaries of CY. After excluding primiparous women (*n* = 990), abortions (*n* = 39) and births in public health facility (*n* = 447), the study population included in this study were 997 multiparous eligible beneficiaries (Fig. 2).

### Operational definitions

The community-based MATIND survey was conducted between July 2013 and November 2014 to capture information from surveyed women who had their recent child birth between January and July 2013. Information on CY utilization and OOPE was also collected for the previous child birth, if any, prior to January- July 2013. We have used the terms ‘subsequent child birth’ (Gave child birth between January and July 2013) and ‘previous child birth’ (Gave child birth prior to January 2013) in the paper to denote the same.

CY utilization was defined as giving child birth in an accredited facility and receipt of either fully or partially subsidized intra-partum care. OOPE was defined as the total expense made by the woman/or her family including pregnancy care cost and other costs related to the birth (treatment related other than pregnancy, transportation, food, and items purchased for the baby). OOPE was expressed in USD ($) after discounting for inflation between year of child birth and 2016.

CY non-utilization comprised three different groups of women: (i) those who did not receive any benefit but delivered in a CY accredited facility, (ii) those who delivered in a non-accredited private facility or (iii) those who gave birth at home.

### Data variables and data collection

Details of data collection in MATIND survey has been described elsewhere [23]. House to house data collection was done by trained research assistants with help of community volunteers. For the descriptive part of the study (objective i and ii), the variables of interest were CY utilization (yes/no) in previous and subsequent child birth; and OOPE in previous child birth. For analytic part of the study (objective iii), CY utilization (yes) in subsequent child birth was outcome of interest. Explanatory variables were socio-demographic characteristics [including age, education (years of study), category of eligibility (BPL and/or ST)], pregnancy related characteristics in previous birth (CY utilization, OOPE, birth outcome) and type of birth (vaginal/caesarean section) in subsequent child birth.

### Analysis and statistics

Data were double-entered and validated in REDCap [24]. The database for this study was imported to EpiData version v2.2.2.183 for descriptive and bivariate inferential analysis (EpiData Association, Odense, Denmark). STATA (version 12.1, copyright 1985–2011 StataCorp LP USA, serial number: 30120504773) was used for regression (Poisson) analysis (enter method).

Frequencies and proportions (categorical variable) were used to summarize CY utilization. The median and interquartile ranges (IQR) were used to summarize the OOPE in the previous child birth. Unadjustedanalyses were performed to assess the association (relative risk, RR) of factors with CY utilization in subsequent child birth. Variables with *p*-value of <0.2 in the unadjusted analysis were included in the regression model. Variables with high collinearity with birth type in subsequent child birth were excluded from the model. Adjusted RRs were reported with 95% confidence intervals (CI).

## Results

### Previous and subsequent CY utilization

Of 997 eligible beneficiaries, 289 (29%) utilized and 708 (71%) did not utilize CY in their previous child birth. Of those who utilized CY (*n* = 289), 182 (63%) utilized CY and 33 (11%) delivered at home in their subsequent child birth. Of those who did not utilize (*n* = 708), 136 (19%) utilized CY in their subsequent child birth. Women with previous CY utilization had three time higher incidence (63% vs. 19%) of subsequent CY utilization. Women with previous CY non-utilization had four times higher incidence (40% vs. 11%) of subsequent child birth at home (Table 1).

**Table 1:** Previous and subsequent CY^a^ utilization among eligible multiparous women who gave child birth between Jan–Jul 2013 in Gujarat^b^, India (*N* = 997)

	Subsequent CY utilization status^c^			
	CY Utilization N (%)	CY non-utilization		
		Pvt. CY accredited	Pvt. Non-accredited	Home
		N (%)	N (%)	N (%)
CY Utilization Status in Previous Child birth^d^				
CY Utilization (*n* = 289)	182 (63)	45 (16)	29 (10)	33 (11)
CY Non-Utilization (*n* = 708)	136 (19)	147 (21)	145 (20)	280 (40)

^a^a state led public private partnership scheme to increase institutional birth among socially disadvantaged population (which includes both schedule tribes and below poverty line); ^b^Study districts- Dahod, Sabarkantha, Surendranagar; ^c^The subsequent child birth of woman with respect to utilization of CY; ^d^The previous child birth of woman with respect to utilization of CY

### Previous OOPE and subsequent CY utilization

Median (IQR) OOPE in previous child birth among those with subsequent CY utilization was $0 ($0,$33) USD and among those with subsequent CY non-utilization was $17 ($0,$49) USD (Table 2).

**Table 2:** Previous OOPE and subsequent CY^a^ utilization among eligible multiparous women who gave child birth between Jan–Jul 2013 in Gujarat^b^, India (*N* = 997)

	OOPE^c^ in previous child birth Median (IQR)
Subsequent CY Utilization^d^	
CY Utilized (*n* = 318)	0 (0–33)
CY Non-Utilization	
Total (*n* = 679)	17 (0–49)
Pvt. CY accredited (*n* = 192)	40 (3–66)
Pvt. Non-accredited (*n* = 174)	49 (11–82)
Home (*n* = 313)	8 (0–20)

^a^a state led public private partnership scheme to increase institutional birth among socially disadvantaged population (which includes both schedule tribes and below poverty line); ^b^Study Districts-Dahod, Sabarkantha, Surendranagar; ^c^Expenses during previous child birth expressed in Median (IQR) in USD; ^d^The subsequent child birth of woman with respect to utilization of CY

### Factors associated with subsequent CY utilization

Previous CY utilization was significantly associated with subsequent CY utilization after adjusting for potential confounders (aRR: 2.7; 95% CI: 2.2,3.3). Zero OOPE in previous child birth was associated with subsequent CY utilization; however, after adjusting for confounders, there was no significant association. Higher Education (secondary or more), belonging to both eligibility categories (BPL and ST) and subsequent vaginal birth were also significantly associated with subsequent CY utilization (Table 3).

**Table 3:** Factors associated^a^with utilization of ‘Chiranjeevi Yojana (CY)’^b^ in subsequent child birth among multiparous eligible women^c^ in Gujarat^d^, India (Jan-July 2013) (*N* = 997)^e^

Variable	Type	Total (N)	CY utilization n (%)	Crude RR (95% CI)	Adjusted RR (95% CI)
Socio-Demographic Variables					
Age					
	≤25years >25 years	461 536	142 (31) 176 (33)	Ref 1.1 (0.9,1.3)	Ref 1.05 (0.9,1.2)
Education					
	No Education Primary Education Secondary Education > High School Education	494 99 301 103	127 (26) 23 (23) 119 (40) 49 (48)	Ref 0.9 (0.6,1.3) 1.5 (1.3,1.9) 1.9 (1.4,2.4)	Ref 0.95 (0.7,1.4) **1.2 (1.1,1.5)** **1.3 (1.2,1.6)**
Eligibility criteria	BPL or ST BPL and ST	726 271	171 (24) 147 (54)	Ref 2.3 (1.9, 2.8)	Ref **1.7 (1.4, 2.1)**
Joint family					
	No Yes	317 680	92 (29) 226 (33)	Ref 1.2 (0.9,1.4)	Ref 1.04 (0.8,1.3)
Pregnancy related Variables					
Previous CY Utilization					
	No Yes	708 289	136 (19) 182 (63)	Ref 3.3 (2.8,3.9)	Ref **2.7 (2.2,3.3)**
Expenditure during Previous Child Birth					
	Expense- No Expense- Yes	370 627	169 (45) 149 (23)	1.9 (1.6, 2.3) Ref	1.04 (0.9,1.3) Ref
Previous Birth Outcome					
	Dead Alive	58 939	13 (22) 305 (32)	Ref 1.5 (0.9, 2.4)	Ref 1.1 (0.7,1.8)
Subsequent Birth Type					
	Operative Vaginal	31 966	2 (6.5) 316 (33)	Ref 5.07 (1.3,19.4)	Ref **3.6 (1.01,12.4)**

^a^regression (poisson), enter method as CY utilization in current child birth as outcome; ^b^a state led public private partnership scheme to increase institutional birth among socially disadvantaged population; ^c^women belonging to socially disadvantaged population; ^d^Dahod, Sabarkantha, Surendranagar; ^e^variables with bivariate *p* < 0.2 shown in table, ^f^Standard of living, Category (BPL/ST/Both) and ANC complications had high collinearity with birth type and were therefore not included in the model

(Odds mentioned as bold are significant values in the model)

## Discussion

Previous CY utilization helped in retaining women within the CY program; there were programmatic significant instances of home births and non-accredited private births despite previous CY utilization. OOPE was zero or relatively low in a great number of previous child births and overall, previous OOPE had no significant effect on the utilization of the scheme in a subsequent child birth.

There were some programmatically relevant findings in our study. *First*, while the utilization of the scheme is low [19], women who utilized CY were more likely to utilize it in the subsequent child birth. The high retention (six out of ten) in the scheme not only indicated a programmatic achievement but also a significant contribution towards the increased trend of institutional birth in Gujarat as found in an earlier study [25].

*Second*, one in ten women who previously utilized CY and four in ten women who previously did not utilize CY gave birth at home. Despite utilizing CY in their previous child birth, this group of mothers gave birth at home for their subsequent birth. The median OOPE in previous births among this group of women was almost negligible (data not shown) and this was intriguing. This is a very important finding that our quantitative analysis could not explore further. Our study was not designed to explore the reason of shifting towards home birth despite utilizing CY in previous child birth.

*Third*, even though previous OOPE was not found to have an effect on subsequent CY utilization, there was some expenditure which has scope for further minimization. This is supported by the finding in our previous study that OOPE among CY beneficiaries was higher than OOPE among women with child birth in public facility [19]. Zero OOPE has been found to be a strong predictor of facility child births in all settings across the globe [2, 7, 26]. The uniqueness of the study was that it has explored previous OOPE (not current) as a factor of utilization; which was not studied earlier [15, 18, 19].

*Fourth*, we found that women who had dual eligibility criteria had higher incidence of CY utilization which is in line with finding of our previous study [19]. Presentation of eligibility documents pertaining to either ST or BPL (any one) might have made them eligible to utilize the CY. In our previous study, “failure to provide the required documentation” was also found as the most common self-reported reason to not utilize CY despite delivering in an accredited private facility [19].

This study had several strengths. This is the first time that a cohort design has been used to study a state led public private partnership scheme’s utilization. Previous experience is known to have a significant effect on the heath belief system of an individual [27]; the same may be applicable to subsequent CY utilization. The data for this study was drawn from one of the largest community-based surveys related to maternal health and about public private partnership scheme in Gujarat; even though it was conducted in only three districts of Gujarat. We used the STROBE checklist to conduct and report the study findings [28, 29]. Data collected was robust as it was double entered and validated to remove data entry errors.

Our study has few limitations. The cohort was in built into data from the community-based survey. Hence, data regarding the previous child birth was collected retrospectively at the time of the survey and recall bias on OOPE cannot be ruled out [30].

### Policy implications

There are some implications for policy and practice. *First*, the CY scheme is performing well in retaining women and reducing OOPE. However, the overall demand side utilization has scope to improve. Steps to increase uptake of CY are expected to increase retention of mothers within CY in their subsequent child birth. In our previous study “failure to provide the required documentation” at the time of child birth was one of the major factors associated with non-utilization [19]. We recommend innovative strategy, say “*e-Chiranjeevi*” to overcome the issue of “failure to provide the required documentation”. This was also recommended in our previous paper [19]. “*e-Chiranjeevi*” may be an extension of the existing Mother & Child Tracking System (eMAMTA) of Gujarat where mother’s information from antenatal registration to child birth to 5 years post child birth is tracked against a unique MAMTA ID. During antenatal registration, the eligibility documentation pertaining to either CY or JSY may be collected and uploaded. These documents may be accessed by the accredited private providers on entering the unique MAMTA ID. This way, the mother may visit a facility of her choice (either public or accredited private) with her unique MAMTA ID. “*E-Chiranjeevi*” by overcoming “failure to provide the required documentation” at child birth may have two fold benefits: i) increasing the utilization of the scheme and ii) retaining mothers within CY in subsequent delivery.

*Second*, systematic qualitative inquiry may be essential to understand both attrition from CY and more importantly the persisting phenomenon of home child birth despite previous CY birth, minimal previous OOPE and substantial financial support in these communities covered by CY. Factors associated with home births have been studied before. However, unknown factors [31, 32] related to home delivery in relation to previous utilization and previous zero OOPE need further systematic enquiry and this may inform specifically designed, targeted interventions to retain mothers in CY.

## Conclusion

Previous CY program utilization resulted in women using the program for their subsequent child birth. However it is essential to address the significant proportion of women who were previously not covered under CY and underwent child birth at home. Systematic qualitative enquiry is recommended to understand why women subsequently delivered at home despite previous CY utilization.

## Appendix 1

**Table 4:** Demographic characteristics of MATIND study districts of Gujarat, India

Indicators^a^	Dahod	Sabarkantha	Surendranagar
Populations (in Millions)	2.1	2.4	1.7
Rural Population (%)	91	85	72
BPL^b^ Population (%)	72	33	47
ST^c^ Population (%)	73	22	01
Literacy Rate (%)	59	65	62
Total Private Providers (No.)	26	83	38
CY^d^-accredited Private Providers (No.)	8	23	21

^a^Size, Growth Rate and distribution of Population. Provisional Population Totals: Census of India-2011 ^b^Population living in Below Poverty Line; ^c^Population belongs to Schedule Tribe; ^d^a state led public private partnership programme ‘Chiranjeevi Yojana (CY)

## Abbreviations

aRR: Adjusted relative risk
BPL: Below poverty line
CI: Confidence interval
C-section: Cesarean section
CY: Chiranjeevi Yojana
eMAMTA: Mother & child tracking system
INR: Indian rupees
IQR: Inter quartile ranges
JSY: Janani Suraksha Yojana
MATIND: Maternal Health India, Research Electronic Data Capture (REDCap)
OOPE: Out-of-pocket expenditures
PPP: Public-private-partnership
ST: Schedule tribe
